# Nitrogen–phosphorus-associated metabolic activities during the development of a cyanobacterial bloom revealed by metatranscriptomics

**DOI:** 10.1038/s41598-019-38481-2

**Published:** 2019-02-21

**Authors:** Jingrang Lu, Bo Zhu, Ian Struewing, Ning Xu, Shunshan Duan

**Affiliations:** 1 U.S. Environmental Protection Agency Office of Research and Development, Cincinnati, OH USA; 2 Institute of Hydrobiology, Jinan University, Guangzhou, Guangdong China; 3 Pegasus Technical Services, Cincinnati, OH USA

## Abstract

The efforts towards reduction of nutrient contamination of surface waters have greatly gained attention to mitigate increasing incidences of harmful cyanobacterial blooms (CyanoHABs), but little attention has been paid on the roles and importance of cyanobacterial N_2_-fixation and phosphorus (P) scavenging pathways during cyanoHABs. Meta-transcriptomic analyses revealed that expressions of genes involved in N_2_-fixation (*nifDKH*) and P-scavenging were significantly upregulated during the bloom compared to pre-bloom in Harsha Lake. The activities of N_2_-fixation occurred during early summer after a late spring phytoplankton bloom, and were associated with high phosphorus and low nitrogen. The highly active cyanobacterial N_2_-fixers were dominated by *Nostoc* and *Anabaena*. Following the activities of N_2_-fixation and production of new nitrogen, an early summer *Microcystis*-dominated bloom, a shift of dominance from *Nostoc* and *Anabaena* to *Microcystis* and an increase of microcystin and saxitoxin occurred. By contrast, P-scavenging activities dominated also by *Nostoc* and *Anabaena* were associated with low P and the *Microcystis* bloom. This information can be used to aid in the understanding the impact that nitrogen and phosphorus have on the early summer CyanoHAB and the functional activities of *Nostoc-* and *Anabaena-*dominated or *Microcystis*-dominated communities, and aid in making management decisions related to harmful algal blooms.

## Introduction

Harmful cyanobacterial blooms (CyanoHABs) are the outcome of eutrophication of water bodies. These blooms can release dangerous levels of cyanotoxins into the affected water bodies. Over the past several decades, CyanoHABs have increased worldwide and represent a serious threat to drinking and recreational water resources, as well as the ecological and economic sustainability of ecosystems^1^.

It has long been known that excessive nutrient (phosphorus and nitrogen) loading results in eutrophication, which can then result in a proliferation of algae, ultimately leading to algal blooms^2,3^. Consequently, CyanoHABs, which typically occur during summer months after phytoplankton succession from diatoms or other low-temperature–adapted algal communities, are a phenomenon that frequently occur with increased nutrient loading from anthropogenic, agricultural, and natural activities in water bodies^4,5^. In Harsha Lake, an early summer *Dinophyta*- and *Chlorophyta*- and *Bacilariophyta-*dominated phytoplankton bloom was observed before detecting a summer *Microcystis*-dominated bloom, and positively associated with nutrients (Supplementary Fig. S1)^4^. It has been commonly adopted that many CyanoHABs are promoted and sustained with exogenous nutrients^6,7^. For example, empirical modeling has shown that both phosphorus and nitrogen are important drivers of cyanobacterial abundance and dominance^8,9^. In assessing how nutrients act on cyanobacterial blooms, most studies have focused on the role of nutrient loading mainly from human activities (quantity of nutrients entering an ecosystem in a given period of time, e.g. nitrogen or phosphorus load to a water body: expressed as tons per year). Considering that cyanobacterial N_2_-fixers are also able to contribute new nitrogen into water bodies through N_2_-fixation, their contribution should also be counted into the nutrient loading. Species of *Nostoc*, *Anabaena Cylindrospermum* and several other genera are widespread in rice fields and contribute significantly to their fertility. For example, in a flooded rice field, N_2_-fixation from N_2_-fixer (*Anabaena* sp. and *Microchaete* sp.) blooms accounted for about 33–41% of nitrogen incorporated^10^. It is believed that the previous studies do not sufficiently explain cyanobacterial community successions and their functions in cyanotoxin production^11^. Recent studies have shown that cyanobacterial N_2_-fixation and phosphorus (P)-scavenging also play important roles in promoting and sustaining CyanoHABs^11,12^. Beversdorf *et al*. (2013) described how a rapid increase of N_2_-fixation formed by dominant *Aphanizomenon* after an early summer declines in nitrogen significantly enhanced N_2_ fixation rates, altered the dominance of cyanobacterial communities from *Aphanizomenon* to *Microcystis* and increased the level of microcystin (MC) in a eutrophic lake (Lake Mendota, Wisconsin, USA)^11^. This study for the first time linked N_2_ fixation to toxic cyanobacteria in freshwater ecosystems and indicated that new nitrogen produced from cyanobacterial N_2_-fixation was an important driver for the dominance of *Microcystis* and microcystin production, but direct evidence of a relationship between a specific cyanobacterial population and N_2_-fixation was not provided. Harke *et al*. observed direct associations of N_2_-fixation (*nifH* gene expression) with dominant cyanobacterial populations of *Anabaena* via an experiment in Lake Erie^12^. In their same metatranscriptomic surveys, the associations of P-scavenging with the specific dominant cyanobacteria *Microcystis* were also observed^12^. In those two studies, the occurrences of N_2_-fixation and P-scavenging, the effect of nitrogen or phosphorus on them and their associations with toxic *Microcystis* dominance were emphasized, respectively. However, a full picture is not so clear about how cyanobacterial N_2_-fixation and P-scavenging occurred, and what their associations with nitrogen and phosphorus, with dominant cyanobacteria, and with cyanotoxin production from pre-bloom to bloom formation were.

Conventional laboratory experiments that add nutrients to cultures in order to investigate the mechanisms of CyanoHABs have limitations that do not allow a complete and precise description of the population dynamic processes of CyanoHAB initiation, maintenance, and termination^13–15^. For example, to address how nitrogen stressed cyanobacteria responses to various nitrogen (nitrate, urea and ammonium) inputs, nitrogen enrichment experiments detected that a *Microcystis aeruginosa* dominated bloom highly responded to ammonium additions, rather than the additions of nitrate and urea, while *Planktothrix agardhii* dominated blooms responded to all the additions of nitrate, ammonium and urea^13^. Next-generation sequencing methods that were established to study bacteria and archaea could also be used to investigate complex ecological phenomena and genomic and transcriptomic characteristics of CyanoHAB-forming species^12,16^. For example, Marchetti and collegease used this method in marine systems to investigate the transcriptional response of diatoms to iron availability^16^.

Considering that there is little information on how varying nitrogen and phosphorus levels impact cyanobacterial metabolic functions, toxic bloom formation, dominance shift, and cyanotoxin production on a short time frame, a short-interval sampling scheme (for example, daily or less than a week prior to and during bloom) is needed. Previously mentioned studies use longer interval sampling schemes, which are weekly sampling in Lake Mendota, Wisconsin^11^ or a one-time fall sampling in Lake Erie^12^, thus they may miss information like metabolic changes or population dominance shift. The meta-transcriptomic analysis of this study was based on the following information: (1) weekly variations of community structures of phytoplankton with emphasis on toxic cyanobacteria^4^, the weekly (May-October) and daily (June) variations of MC and MC-producers using qPCR and RT-qPCR [Lu *et al*. submitted], and dominant cyanobacterial compositions based on 16S rRNA Illumina sequencing from May to October^17^. This novel approach in intensive sampling and comprehensive community analysis provided us complete information of microbial community structures and successions and enabled us to select samples representative of different blooming stages. Under such experimental design, we examined: (1) the changes of global gene-expressions between the pre-bloom and during-bloom periods, and (2) the associations of those upregulated genes with the levels of nitrogen and phosphorus, and with to dominant cyanobacterial abundance and production of cyanotoxin in a eutrophic lake in Ohio.

## Results

### Transcriptomic analysis of the overall microbial community and functional categories

A total of 15,899,228 reads of high-quality sequences with an average sequence length of 199.02 ± 75.64 (standard deviation) bp and GC content of 47.47 ± 6.71% were retrieved. Library recoveries varied from 21,326 to 499,108 reads, with an average of 274,124 reads (Table S2). Of 741,631 reads of predicted protein features, 277,015 reads (37.35%) were annotated. Of the four retrieved domains, Bacteria constituted the major part (relative abundance [RA], 69.34%), followed by Eukaryota (RA, 30.54%), Archaea (RA, 0.07%), and Viruses (RA, 0.06%). Of the characterized Bacteria (28 phyla), phylum Cyanobacteria (RA, 62.86%) dominated the community, followed by Proteobacteria (2.87%) and Bacteroidetes (1.35%). Firmicutes made up 0.75% of the reads and Actinobacteria made up 0.60%.

**Figure 1:**
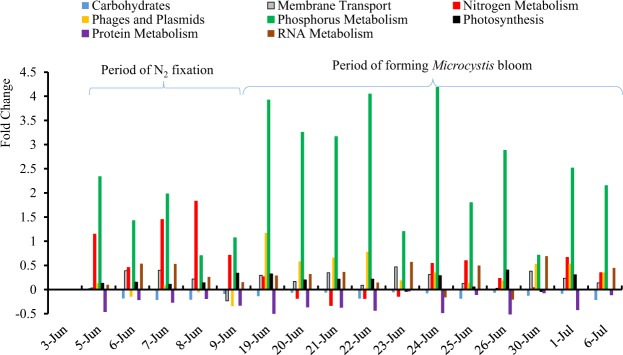
Fold changes of the major functional categories from June 3 (pre-bloom date) to July 6.

Annotated genes from our transcriptomic analysis fell into 28 functional categories at the top level of Gene Ontology (GO). Genes involved in photosynthesis (36%) were the most abundant, followed by 10 other functional groups with >1% of the proportion, including protein metabolism (13%); carbohydrates (9%); respiration (7%); clustering-based system (7%); cofactors, vitamins, prosthetic groups, and pigments (4%); miscellaneous (4%); phages, prophages, transposable elements, and plasmids (3%); RNA metabolism (3%); amino acids and derivatives (3%); and stress response (2%). Nitrogen and phosphorus metabolisms were among the 10 functional categories, representing approximately 1% of the total annotated sequences. The remaining categories were <1% of the total. To examine temporal gene expression patterns of these gene groups, we used the expression level of genes on June 3 as a reference point to calculate relative fold change of genes collected at all the following times. Pathways that were highly expressed included phosphorus, nitrogen, photosynthesis, membrane transport, and phage, while genes involved in protein metabolism had low expression levels (Figs 1 and S2). We found positive correlations between protein and RNA metabolisms (R^2^, 0.619) and between photosynthesis and phosphorus metabolisms (R^2^, 0.559), while negative correlations were observed between photosynthesis metabolism and protein metabolism (R^2^, −0.884) and RNA metabolism (R^2^, −0.662) (Supplementary Fig. S2). Based on the fold-change analysis, metabolic pathways for membrane transport, nitrogen, phage, photosynthesis, phosphorus, and RNA were found to be positive or increased over time as compared to June 3. Among these, the positive fold changes in gene expression, which were higher than the average fold change (0.36 folds), were phosphorus metabolisms (2.33 folds) and nitrogen metabolism (0.47 folds) (Fig. 1).

### Nitrogen-related genes in Cyanobacteria

**Figure 2:**
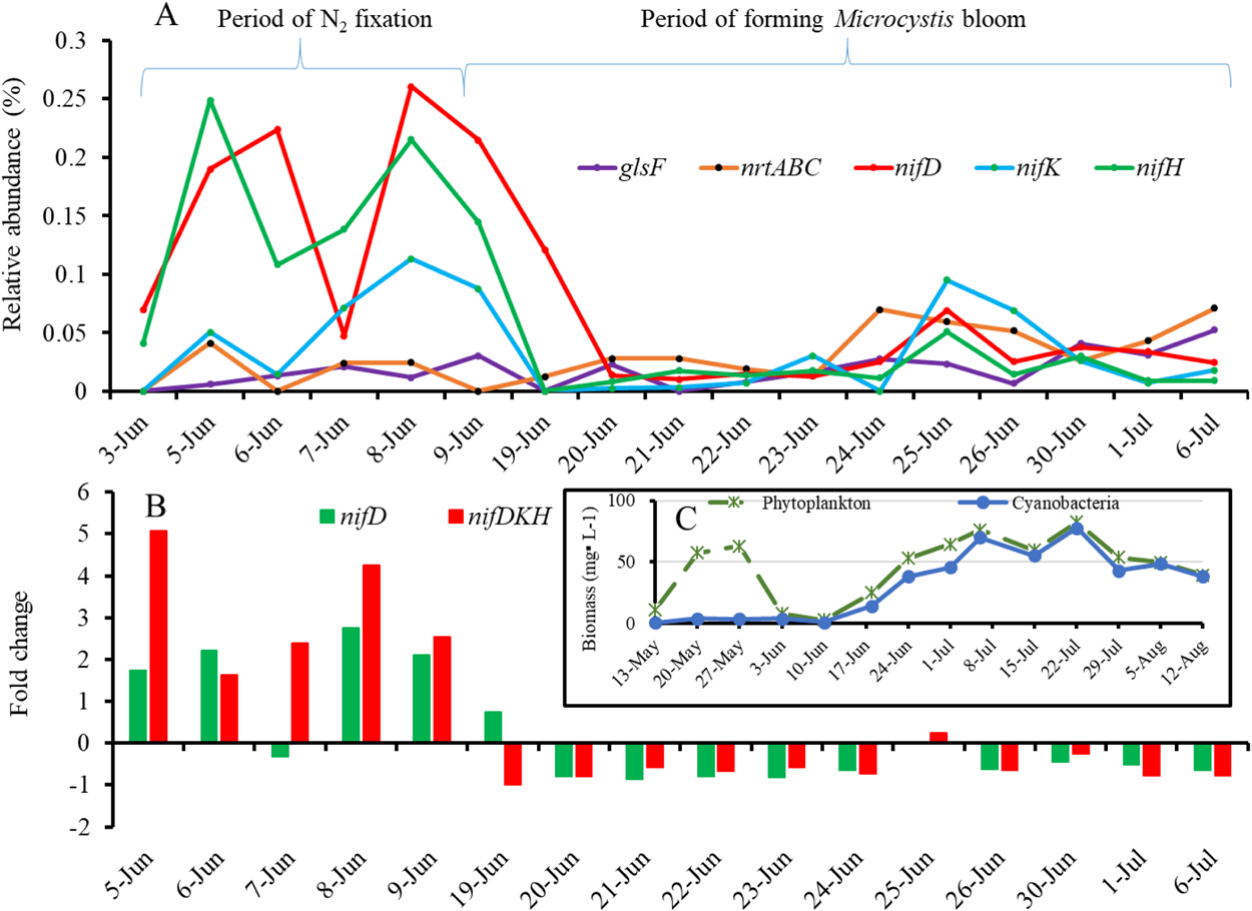
Variations of (**A**) relative abundance of nitrogen-related genes, (**B**) fold change of N_2_-fixing associated genes (*nifD* and *nifDKH*), and (**C**) biomass of phytoplankton and cyanobacteria used as a reference of the N_2_-fixation activities (arrow) found in this study relative to phytoplankton bloom (pre-June 3) and cyanobacterial bloom (post-June 3). Arrow indicate peak *nifDKH* expression levels.

Analyses of nitrogen-related genes during pre- and bloom-periods revealed the upregulation of nitrogenase FeMo alpha- and beta-chains (*nifD* and *nifK*), and FeMo reductase and maturation protein (*nifH*), but not the ferredoxin-dependent glutamate synthase (*glsF*) and ATP-binding protein of nitrate ABC transporter (*nrtA*, *nrtB*, *nrtC*) (Fig. 2A). The three nitrogenase genes (*nifD*, *nifK*, and *nifH*, or *nifDKH*) showed peak expression levels from June 5 to 9, which coincided with the end of the *Dinophyta*-, *Chlorophyta*- and *Bacilariophyta*-dominated phytoplankton bloom^4^ (Fig. 2B,C), the significant increase of TNH4 and TN, and with the initial stages of a *Microcystis* bloom. The expression levels of *nifDKH* genes decreased and remained at a low level after June 19, when the TNH4 started to fall after reaching a peak and the *Microcystis* bloom continue to develop (Fig. 2C arrow). Compared to the initial expression level, *nifD* increased 0.74–2.75 folds, while *nifH* increased 1.64–5.07 folds from June 5 to June 9 (Fig. 2B), with both changes in expression levels being significant (Wilcoxon test, P_*nifD*_ = 0.005 and P_*nifH*_ = 0.004, respectively). In the meanwhile, compared to the average values of whole samples, total ammonium increased 1.65 times from June 5 to July 6 (P value of t-test between two means: <0.0001) and peaked on June 21, while total nitrogen increased by 1.24 times from June 10 to July 6 (P value of t-test between two means: 0.01) and peaked on July 6 after the N_2_-fixation peak (Supplementary Fig. S3), suggesting that a new nitrogen source was added to the water after the N_2_-fixation process occurred. There were no noticeable changes in *glsF* and *nrtABC* expression (Fig. 2A), which are genes associated with ammonium incorporation into carbon skeletons (*glsF*)^18^ and assimilation of nitrate or nitrite (*nrtABC*)^19^. Corresponding to the *glsF* and *nrtABC* gene activities, TNO3 and TNO2 were in a very low level in June (Supplementary Fig. S3).

**Figure 3:**
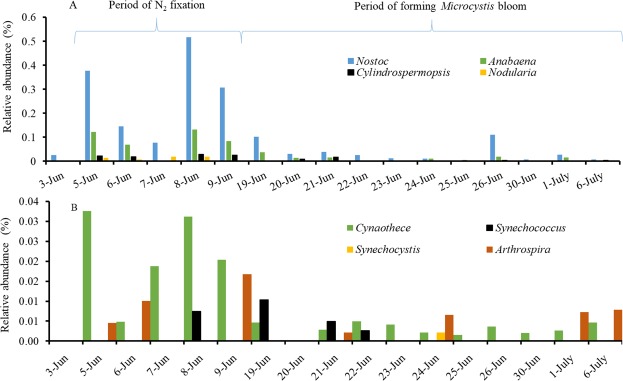
Variation in the relative abundances of N_2_-fixing associated-like sequences in (**A**) *Nostocales* (*Nostoc*, *Anabaena*, *Nodularia*, and *Cylindrospermopsis*), (**B**) *Chroococcales* (*Synechococcus* and *Synechocystis*) and *Oscillatoriales* (*Cyanothece* and *Arthrospira*) taxonomic groups.

Next, we examined the abundance of expressed nitrogen-related genes within the identified taxa. The sequences similar to *nifDKH* genes associated with two types of N_2_-fixers (Supplementary Table S3): heterocystous isolates from *Nostocales* (*Nostoc*, *Anabaena*, *Nodularia*, and *Cylindrospermopsis*) (Fig. 3A) and non-heterocystous isolates from *Chroococcales* (*Synechococcus*, *Microcystis*, and *Synechocystis*) and *Oscillatoriales* (*Cyanothece*, *Arthrospira*, and *Trichodesmium* or *Planktothrix*) (Fig. 3B). Specifically, a majority of the sequences were mapped to *Nostoc* (RA, 0.066%) and *Anabaena* (RA, 0.019%), followed by *Cylindrospermopsis* (RA, 0.005%), *Cyanothece* (RA, 0.005%), *Nodularia* (RA, 0.003%), *Arthrospira* (RA, 0.002%), *Synechococcus* (RA, 0.001%), and others with an RA of <0.001%. For the last group including *Microcystis*, *Synechocystis*, and *Trichodesmium* or *Planktothrix*, there is limited or even no reference to support that they are capable of N_2_-fixation. Further correlation analysis between total *nifDKH* and *nifDKH* expressed genera showed that the major N_2_-fixers were *Nostoc* (R^2^, 0.914; *P* < 0.0001), *Anabaena* (R^2^, 0.903; *P* < 0.0001), *Cylindrospermopsis* (R^2^, 0.834; *P* < 0.0001), *Nodularia* (R^2^, 0.709; *P* < 0.0001) and *Cyanothece* (R^2^, 0.874; *P* < 0.0001). In addition, a number of genera of active N_2_-fixers, especially within *Nostocales*, were correlated with each other (Supplementary Table S4). All of these active N_2_-fixers increased during the same period (early June). The result suggested that *Nostocales* were the main driver of the community wide N_2_-fixation, and indicated that the change in expression of N_2_-fixation genes co-varied with changes in those active N_2_-fixating *Nostacales*.

**Figure 4:**
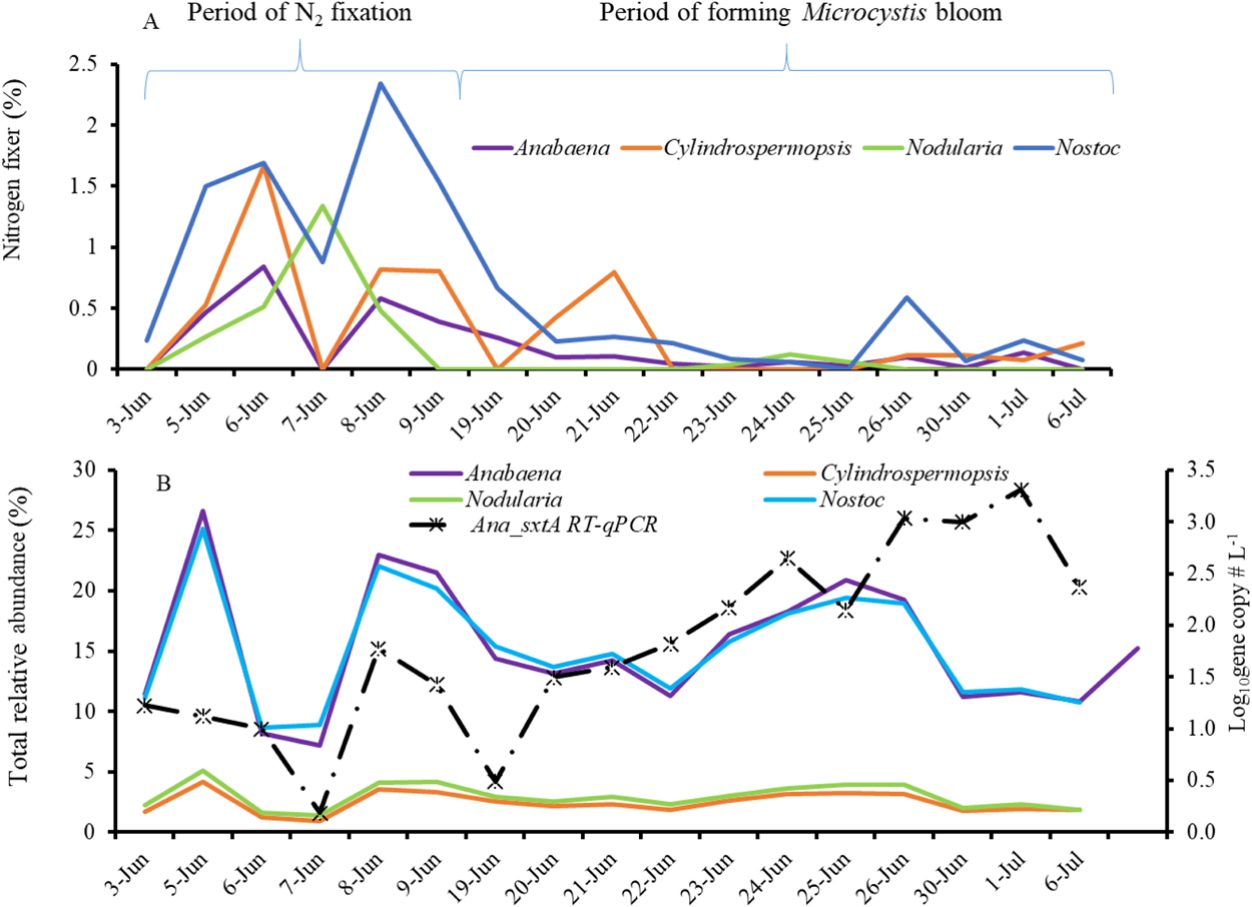
Relative abundance of active N_2_-fixers within *Nostocales* (**A**) and total relative abundance of each major genus, and the *sxtA* gene transcripts as measured by RT-qPCR (**B**).

**Figure 5:**
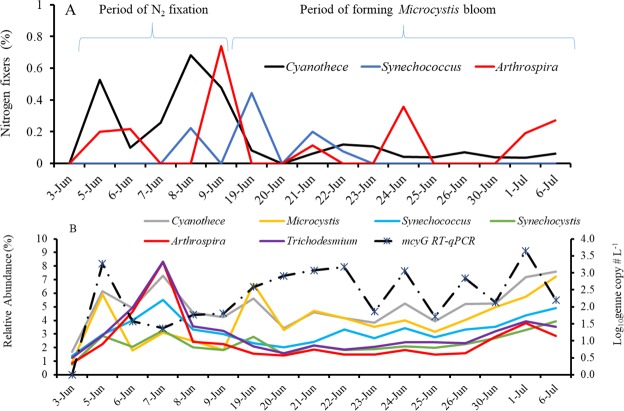
Relative abundance of active N_2_-fixers within *Chroococcales* and *Oscillatoriales* (**A**) and total *Chroococcales* and *Oscillatoriales* of each major genus and the *mcyG* gene transcripts as measured by RT-qPCR (**B**).

Figures 4 and 5 illustrate the proportions of the active N_2_-fixers among the corresponding general taxa. Specifically, the RA of *nifDKH*-upregulated *Nostocales* was 0.219% of the total RA of *Nostocales*, while the RA of *nifDKH*-upregulated *Chroococcales* and *Oscillatoriales* was only 0.151% of the total RA within the two orders, indicating higher proportions of active N_2_-fixers in *Nostocales* relative to others. The percentages were also significantly higher from June 5 to June 9 than at other times (*P*_*Nostocales*_ = 0.003, *P*_*Chroococcales-Oscillatoriales*_ = 0.030) (Figs 4A and 5A). Although those total populations occurred in high RA during the entire investigation (Figs 4B and 5B), the upregulated expressions of N_2_-fixation genes were only observed during the period with high proportions of active N_2_-fixers.

To examine the association of N_2_-fixation with cyanotoxin production, the transcripts of MC and saxitoxin producing gene (*mcyG* and *sxtA*) were detected from the total RNA of each sample. In the meanwhile, MC and saxitoxin were also detected (Supplementary Fig. S1). The gene expressions for both cyanotoxins were high after June 19 when the RA of active N_2_-fixers declined. The increasing RAs of total *Nostoc* and *Anabaena* agreed with those in the *Anabaena*- and *Nostoc*-specific *sxtA* gene transcripts, which corresponded to the high saxitoxin measurements in early June (Fig. 4). Similarly, the increasing RAs of total *Microcystis* agreed with those in the *Microcystis*-specific *mcyG* gene transcripts that corresponded to the high MC measurements in early June (Fig. 5).

**Figure 6:**
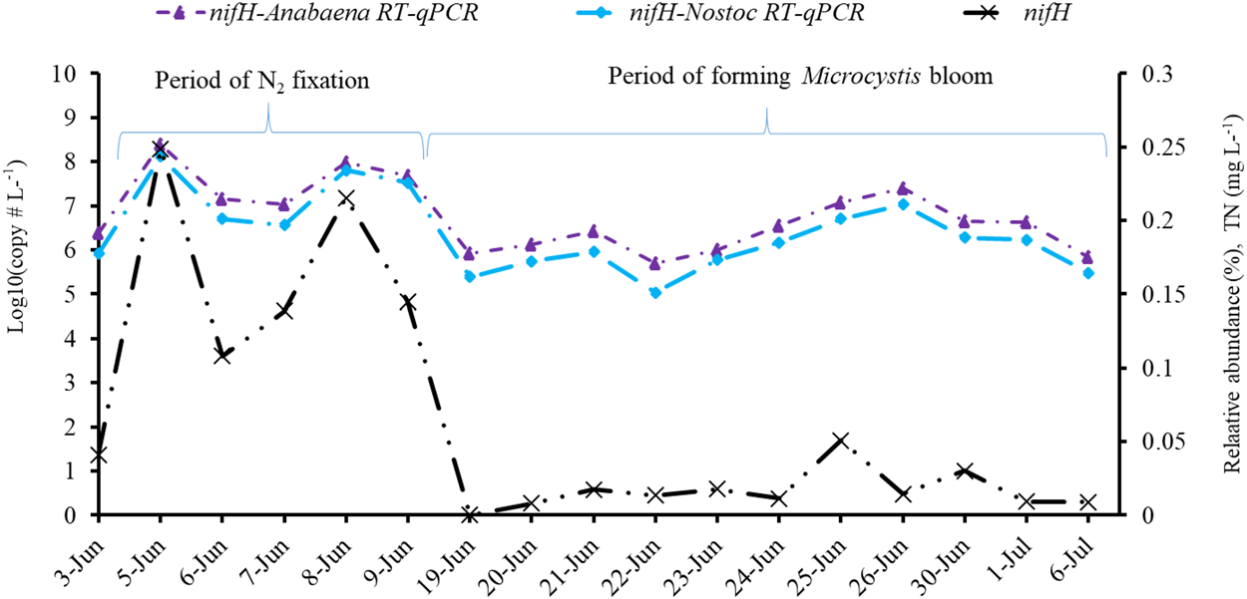
*Nif* gene expressions in *Anabaena* (*nifH-Anabaena* RT-qPCR) and *Nostoc* (*nifH-Nostoc* RT-qPCR) during the early stages of cyanobacterial bloom.

To specifically determine if *nifH* gene expression found in the transcriptomes are associated with *Anabaena* and *Nostoc*, a *nifH* RT-qPCR assay specifically detecting this gene in both *Anabaena* and *Nostoc* was performed (Fig. 6). Results showed that both *Anabaena*- and *Nostoc*-specific *nifH* transcript numbers were consistent with *nifH* RAs, and the peaks of *nifH* RAs corresponded with those of the two RT-qPCR signals during June 5 to June 9. These peak expressions corresponded to the peak RA of *Nostoc* and *Anabaena* (Figs 2 and 3).

### Phosphorus functional gene transcriptions in different organisms

**Figure 7:**
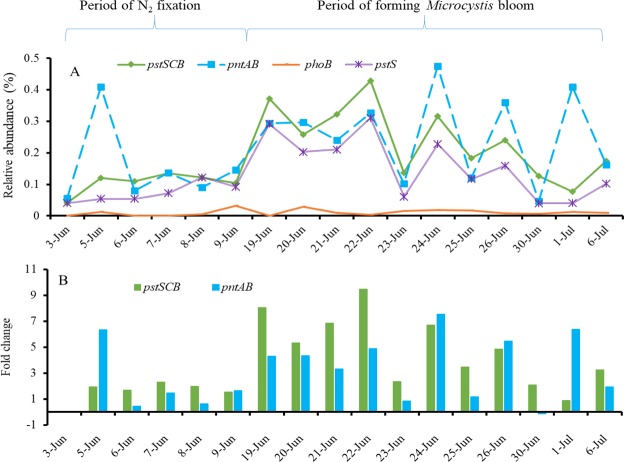
Variations of (**A**) relative abundance of phosphorus-related genes, (**B**) fold change of phosphorus associated genes (*pstSCB*).

Expression of genes associated with the phosphorus metabolic pathway were also observed (Fig. 7). The major genes were those associated with phosphorus scavenging, including phosphate-binding protein (*pstS*), phosphate import ATP-binding protein (*pstB*), phosphate transport system permease protein (*pstC*), and phosphate regulon of transcriptional regulatory protein (*phoB*: positive regulator for phosphorus assimilation when phosphate is limited), which are considered to be involved in phosphorus accumulation under limited phosphate conditions in cyanobacteria^12,20,21^. *PstS* showed the highest upregulated expression among *pst* genes (*pstSCB*) accompanied by the other two genes (*pstCB*) at a lower level but a similar trend (data shown for relative abundances of each of the three genes in the supplementary spreadsheet). When expressions of these genes were evaluated during the time periods examined in this study, the combined RA of *pstS*, *pstC*, and *pstB*, or p*stSCB*, increased by 1–9 folds relative to the pre-bloom date (Fig. 7B). Moreover, expression levels were at their lowest level during the high N_2_-fixation period (June 5–9). The highest level was from June 19 to June 22, with expression levels going down again in late June (Fig. 7). The other highly upregulated genes were NAD(P) transhydrogenase α subunit (*pntA*) and PhosNAD(P) transhydrogenase β subunit (*pntB*), which catalyzes redox reaction and mediates energy-dependent reduction of NADP with NADH by using the electrochemical proton gradient as the driving force, providing reduction power for biosynthesis^22^. The *pntAB* transcript also showed high relative abundance and displayed a similar trend to that of *pstSCB* during most of June (except on June 5 when the initial N_2_-fixation occurred), increasing by 0–7 folds from the pre-bloom date (Fig. 7).

The primary order of taxa associated with the highly expressed phosphorus metabolic pathway-associated genes were *Nostocales* as found in nitrogen metabolism. The relative abundances of phosphorus related genes from *Nostocales* were 4.5 times higher than those from the other two orders (*Chroococcales* and *Oscillatoriales*) (RA, 0.003%) (Supplementary Fig. S4). As with N_2_-fixation, relative abundances among different genera associated with phosphorus metabolism were significantly correlated, especially within *Nostocales* (Supplementary Table S2).

**Figure 8:**
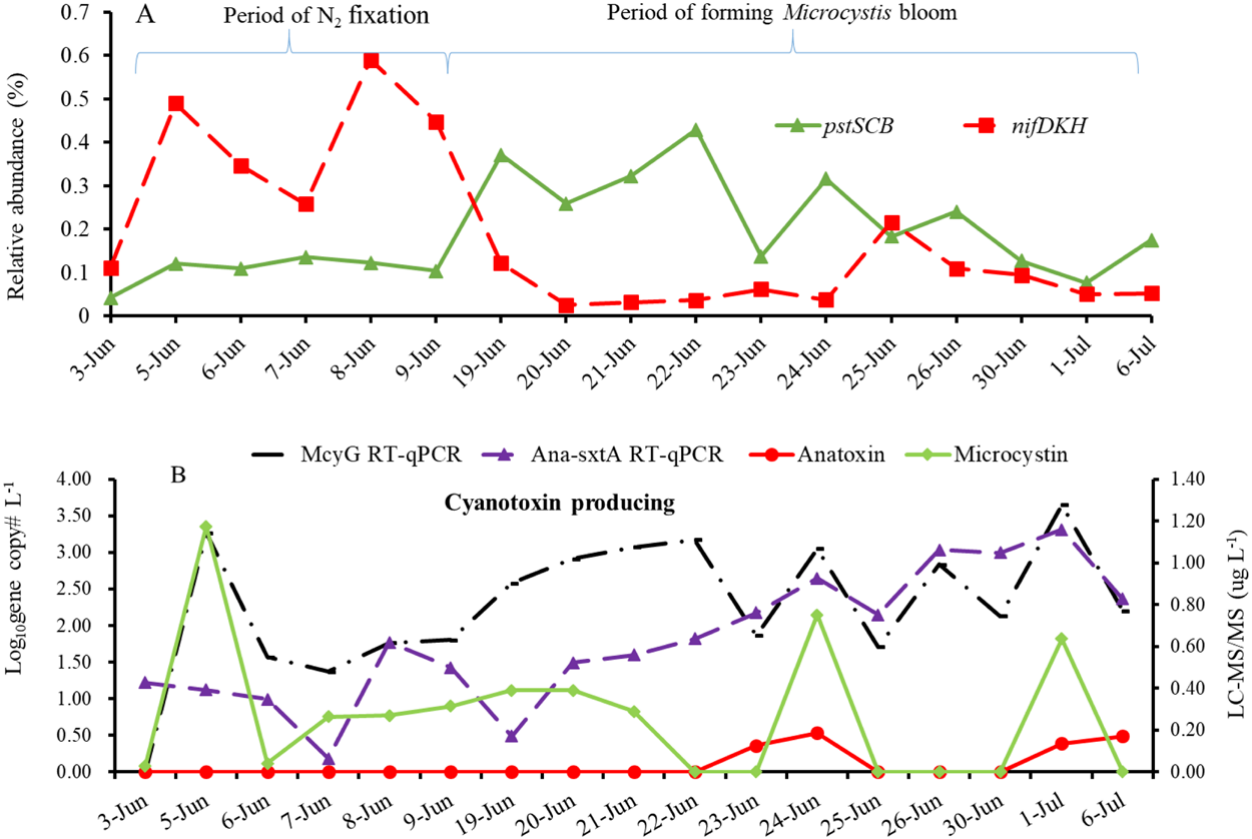
Associations of (**A**) N_2_-fixation (*nifDKH*) and phosphorus scavenging (*pstSCB*) with (**B**) cyanotoxin producing indicated by toxic *Microcystis* qPCR (*mcyG*) and toxic *Anabaena* qPCR (Ana-sxtA) during a CyanoHAB forming.

RT-qPCR analysis of *pstS* gene transcription showed a trend similar to the relative abundance (R^2^, 0.25), as copy numbers were lower in early June and higher in mid-June (Fig. S5). Notably, the RA of *pstS* was negatively correlated with total phosphorus levels (R^2^_RA-*pstS*_, −0.78) (Fig. 8A,B), indicating that a phosphate accumulating process was occurring under phosphate limitation or starvation.

## Discussion

This study found that nitrogen and phosphorus metabolisms were the top two categories to increase their gene expressions prior to and during a toxic algal bloom. The findings provided the evidence that genes associated with nitrogen and phosphorus metabolisms played important roles in cyanobacterial bloom formation. It has been assumed that cyanobacterial blooms are a consequence of a synergistic interaction between available nutrients and the organisms’ physiological capabilities to use such nutrients under favorable weather conditions^7,23,24^. Among the nutrients, nitrogen and phosphorus are two of the most important bloom drivers^6,23,25–27^. Here, we examined the genes and cyanobacterial populations involved in nitrogen and phosphorus utilization before and during a bloom in Harsha Lake, OH. Our study found the associations of upregulation of nitrogen and phosphorus associated genes with dominant populations and their metabolic activities, when nitrogen or phosphorus levels changed during a toxic algal bloom event. The new findings of this study were that two biological processes, N_2_-fixation and P-scavenging, led by the dominant *Nostocales* order played important roles during a CyanoHAB event.

The low nitrogen level that occurred in early June might have been caused by overgrowth of *Dinophyta*, *Chlorophyta* and *Bacilariophyta* from May 13 to May 27. During that time, their biomasses reached up to 45, 11, and 9 mg L^−1^, respectively (Supplementary Fig. S1A)^4^. The co-occurrence of cyanobacterial N_2_-fixers and their N_2_-fixation metabolites with low nitrogen level suggests that the decreased nitrogen level could be a factor that caused the community successions from an early spring eukaryotic algal bloom to the multiplication of those *Nostocales* with heterocysts, which are able to use N_2_ by N_2_-fixation. For example, the biomass of *Anabaena* was increased stably from no detection on May 13 to 1.2 mg L^−1^ on June 3^4^. It has been shown in both transcriptomic profiles (this study) and microscopic counts^4^ that *Nostocales* presented as the major group throughout the cyanobacterial bloom in Harsha Lake. *Nostocales*, which includes *Anabaena*, *Aphanizomenon flos-aquae*, and *Nostoc*, are able to fix nitrogen through heterocysts and are known to be a major group of N_2_-fixers^28^. For those nitrogenase-similar sequences hitting non-heterocyst cyanobacterial genomes, *Arthrospira maxima* CS-328, *Cyanothece* sp. PCC 7424/7425/8801/8802, *Trichodesmium erythraeum* IMS101, *Synechocystis* sp. PCC 6803 and *Synechococcus* sp. JA are the isolates (Table S3) found to be capable of N_2_-fixation^29^ through diazotrophic activity by photosynthesizing during the day and N_2_-fixing at night^30^. Of the non-heterocyst isolates hit with some nitrogenase-similar sequences (Table S3), *Microcystis aeruginosa* has not been proven to be a N_2_-fixer and further evidence is needed to find whether those related transcripts are involved in N_2_-fixation. The nitrogenase-similar sequences hitting non-heterocyst cyanobacterial genomes were less abundant and only accounted for 8.4% of the total active N_2_-fixers in this study. We believe that the non-heterocyst cyanobacteria, especially *Microcystis*, could be insignificant or have no contribution to N_2_-fixation based on their proportions of expressed N_2_-fixation genes. In agreement with previous findings^11^, the N_2_-fixation activity in our study was greatest in early summer (June 3–June 20) and considered to be an N_2_-fixation event, when N_2_-fixers were dominant and nitrogen levels were low. Beversdorf and colleagues^11^ observed the first major N_2_-fixation event in early summer to be comprised mostly of the N_2_-fixer *Aphanizomenon*, possibly due to short-term nitrogen stress and/or limitation. We found that the proportion of those *Nostocales* (mainly *Anabaena* and *Nostoc*) with upregulated nitrogen-related genes should be noted for potential occurrence of N_2_-fixation.

Up to this study, there have been multiple lines of evidence demonstrating a dominance shift from an N_2_-fixer–dominated community to a non-N_2_-fixer–dominated community. The evidence observed using microscopy was that *Anabaena* occurred from mid-May following an early summer phytoplankton peak and trended to increase and be dominant in the cyanobacterial community until late-June, while *Microcystis* occurred in early-June and started to be dominant from late-June until early-August (Supplementary Fig. S1A)^4^. The following 16 S rRNA Illumina sequencing data showed the dominance of *Anabaena* early to mid-June, and the successive dominance of *Microcystis* from late June to early August (Supplementary Fig. 1B). Lastly, the qPCR data not only confirmed those cyanobacterial community data, but also clearly showed the shift of dominance from the *Anabaena* to MC-producing *Microcystis* (Fig. 1C). This study provided further evidence that the dominant *Anabaena* observed^4^ should be the potential N_2_-fixers with upregulated nitrogen-related genes from early- to mid-June.

Thus, our findings were in agreement with the previous study^11^: N_2_-fixation in the early summer stimulated and extended a bloom of non-N_2_ fixing cyanobacteria, dominated by *Microcystis*, based on measurement of N_2_-fixation rates. Here, the “non-N_2_-fixing cyanobacteria” should be described as inactive populations of N_2_-fixation based on our results (Figs 4 and 5). As suggested by Beversdorf *et al*., a consequence of the stimulation of N_2_-fixation was cyanobacterial community succession, formation of another community structure and increase of cyanotoxin producing productions. In our study, growth of the *Microcystis* population started in late June, after the N_2_-fixation event, and lasted through July^4^. Thus, cyanobacterial N_2_-fixation could play an important role in summer cyanobacterial blooms in lakes that are under lower nitrogen level in the early summer by the addition of new nitrogen to the water body (Fig. S3).

It has been observed that transcripts of genes related to phosphorus scavenging (for example, *pstS*, *C*, *B*, *A*, and *pstSCBA*) and assimilation (*pho* regulon) are upregulated during phosphorus depletion in cultures of *Anabaena* sp.^12,21^. In this study, the relative abundance of *pstS* was negatively correlated with TP concentration (R^2^, −0.78, *P* = 0.0013) and total reactive phosphate concentration (R^2^, −0.49, *P* = 0.045). The low phosphorus level from June 20 to June 30 could be a phosphorus starvation period, during which *Microcystis* continued to increase and *pstS* expression by *Nostoc* and *Anabaena* also increased. This result is consistent with previous results that indicated *pstS* is always induced under phosphate starvation conditions^12,20,21^. Furthermore, this study revealed that *Nostoc* and *Anabaena* were the major players in both N_2_-fixation and P-scavenging under nitrogen and phosphorus stress. As for the highly upregulated *pntAB*, their variations that were consistent primarily with those of *pstSCB*, indicated that *pstSCB* expressions were involved with active photosynthesis^31,32^ along with the interconversion between NADP(H) and NAD(H). These gene products allow the interconversion of NADP(H) to NAD(H), and their overexpression may lower the normally high NADP(H)/NAD(H) ratio present during active photosynthesis^31,32^. In addition to the associations among phosphorus levels, phosphorus metabolism, and a *Microcystis* bloom, there seemed to be associations between phosphorus and N_2_-fixation. Initiation and development of N_2_-fixation was associated with higher phosphorus levels and *nifDKH* expression but lower nitrogen level and *pstSCBA* expression, while the opposite was observed as N_2_-fixation declined. Specifically, the relative abundance of *pstSCBA* genes and nitrogen was low from June 3 to June 9 and then rose after June 19; this contrasts to the trend for phosphorus and the *nifDKH* gene expression. This result agrees with meta-transcriptomic surveys and observations of a *Cylindrospermopsis* bloom in other lakes^12,30^ where high phosphorus conditions promoted N_2_-fixation gene (*nifH*) expression in *Anabaena*, while low phosphorus conditions promoted the expression of *pstSCAB* and *phoX* for phosphorus accumulation in *Microcystis*. Considering the important role of active cyanobacterial N_2_-fixation and P-scavenging, highly abundant transcripts represented by *nifH* may act as indicators of low level of nitrogen and dominance of active *Anabaena*-*Nostoc* N_2_-fixers, while the high signal of *pstS* RT-qPCR may indicate the need for or depletion of phophorous. Both could act as predicators for cyanotoxin production, as indicated in Figs 8 and S5.

Collectively, the significantly upregulated N_2_-fixation gene *nifDKH* and phosphorus metabolic genes *pstSCB*, *pntAB*, and *phoB*, which indicate significant activities of N_2_-fixation and P-scavenging, involved with *Nostoc* and *Anabaena* dominated communities, were revealed by global transcriptomic analysis. The activities of N_2_-fixation were associated with high phosphorus, low nitrogen, and high relative abundance of active cyanobacterial N_2_-fixers, while those of P-scavenging were associated with decreasing phosphorus and increasing *Microcystis*. The observed *Microcystis*-dominated summer bloom was promoted by N_2_-fixation activities of N_2_-fixers *Nostoc* and *Anabaena*. The development of the bloom might be sustained by newly added external nitrogen through N_2_-fixation and by accumulated internal phosphorus through P-scavenging. Thus, the outcome was the shift of cyanobacteria community from *Nostoc* and *Anabaena* dominated one to *Microcystis* dominated one and the increase of cyanotoxin production. This information can be used to aid in the understanding the impact that nitrogen and phosphorus have on cyanobacterial community successions and cyanotoxin production and aid in making management decisions related to harmful algal blooms.

## Methods

### Harsha Lake, sample processing and quality control

Harsha Lake is an agricultural impacted inland lake, 25 miles east of Cincinnati in southwestern Ohio. The East Fork drains an area of 499 mi^2^ before it flows into the Little Miami River. Harsha Lake was created when the East Fork was impounded in 1978. The total watershed area that drains into Harsha Lake from the East Fork drainage basin is approximately 344 mi^2^. The drainage basin upstream from Harsha Lake is mainly agriculture area, while urban areas and forests dominate around the lake and downstream from Harsha Lake dam (https://pubs.usgs.gov/wri/wri034216/). The location, sampling, and processing of samples have been described previously^4^. For this study, samples from 17 sampling dates collected during CyanoHAB-forming (from June 3 to July 6) were used for the cyanobacterial transcriptomic analysis. As references, additional samples (weekly samples from May 6 through September 30, and daily samples from June 5 to June 26) were mentioned. Those samples were employed to examine the community structures of phytoplankton with emphasis on toxic cyanobacteria (weekly samples)^4^, the role of qPCR and RT-qPCR for monitoring variations of microcystin producers and early warning their toxin production (daily and weekly samples) [Lu et al. submitted], and the interspecific and intraspecific diversity and interaction of cyanobacteria throughout a bloom^17^. Using a Durapore polyvinylidene fluoride (PVDF) filter (0.45 μm, MilliPore, Foster City, CA),100–200 mL of water was filtered. Two replicate filters were made from each sampling site studied on a fixed buoy in Harsha Lake. Each filter was then inserted into 1.5-mL microtube that contained a 600 μL RLT plus solution with RNase inhibitor (QIAGEN, Valencia, CA) and stored at −80 °C until RNA extraction. The filter was disrupted and lysed using a Mini-Beadbeater-16 (BioSpec Products, Inc., Bartlesville, OK) twice for 30 sec and then centrifuged at 10,000 *g* for 3 min. The supernatant was then transferred to a new sterile tube, and RNA was extracted and purified using an AllPrep RNA kit (QIAGEN) following the manufacturer’s instructions. The extracted RNA was eluted in 50 µL RNase-free water (Sigma-Aldrich, St. Louis, MO), and genomic DNA was removed using the TURBO DNA-free kit (Life Technologies, Foster City, CA). The RNA concentration was estimated with a Nanodrop ND-1000 spectrophotometer (NanoDrop Technologies, Inc., Wilmington, DE). RNA quality was assessed using electrophoresis on an Agilent 2100 bioanalyzer (Agilent Tech. Inc., Santa Clara, CA). The DNA-free RNA was stored at −80 °C until used.

### RNA sequencing

For the RNA sequencing (RNA-Seq) in this study, samples (17 days × 2 replicates) were used from June 3 to July 6, 2015. This experimental design was based on the observed results of cyanobacterial counts (Supplementary Fig. S1a) by microscopy^4^, on the cyanobacterial genus compositions using rRNA gene Illumina sequencing (Supplementary Fig. S1B), on genomic copy numbers using qPCR targeting MC-producer specific *mcyE*, toxic *Microcystis*-specific *mcyG*, saxitoxin producer -specific *sxtA* (Supplementary Fig. S1C), anatoxin producer-specific *anaC* and cylindrospermopsin producer-specific *cyrA*, and MC measurements using. Liquid Chromatography Mass Spectrometry (LC-MS/MS) (Supplementary Fig. S1B). Microscopic counts and qPCR results for cyanotoxin producing populations indicated that an observed cyanobacterial bloom started in early June, while MC was detected just after June 3. Therefore, June 3 was defined as the beginning of the CyanoHAB, or pre-bloom date, and dates after June 3 were defined as the during-bloom dates. For each sample, approximately 200 ng of DNA-free RNA was treated with Ribo-Zero (Illumina, San Diego, CA) to remove ribosomal RNA and then purified using the RNeasy kit (QIAGEN). cDNA synthesis was performed using the SuperScript double-strand cDNA synthesis kit (Life Technologies) according to the manufacturer’s instructions. The cDNA was purified and cDNA libraries were prepared following the manufacturer’s protocol using an Illumina NexteraXT DNA library prep kit (Illumina). The quality and size spectra of cDNA libraries were examined using a Bioanalyzer (Agilent 2100, Agilent Tech. Inc.). Sequencing was performed and data were generated from paired end reads (2 × 300 bp) on a MiSeq system (Illumina). The base-calling pipeline (Illumina NexteraXT) was used to process the raw fluorescence images and call sequences. Raw reads with >10% unknown nucleotides or with >50% low-quality nucleotides (quality value <20) were discarded.

### Sequence data analyses

Taxonomic mapping and gene function annotation were conducted with the Meta Genome Rapid Annotation using Subsystem Technology (MG-RAST v3.3), server at the Argonne National Laboratory (http://metagenomics.anl.gov) according to the laboratory’s manual. It incorporates several databases including National Center for Biotechnology Information-nr, ResSeq, IMG, Kyoto Encyclopedia of Genes and Genomes, COG, SEED, and Subsystems. The RNA-Seq dataset (submission number SRP127716) was analyzed primarily using the Subsystems database, which was set the cut-off values of a maximum e-value of 1e-5, a minimum identity of 60% and a minimum alignment length of 15 measured in amino acid for protein and base pair for RNA databases^33^. The MG-RAST v3 annotation pipeline uses the steps to map one read to multiple annotations and one annotation to multiple reads through three steps: (1) not one-to-one gene prediction step, (2) clustering identified sequence at 90% amino acid identity and performing one search for each cluster and (3) the not one-to-one annotation process itself^33^. Relative abundance (RA; %) was calculated as the percentage of the retrieved sequence numbers for each category divided by total sequences in each library. To identify the main cyanobacteria involved in nitrogen and phosphorus metabolisms, the number of cDNA sequences that mapped to each cyanobacterial species or above taxonomies associated with the predicted nitrogen and phosphorus metabolic genes were retrieved. The relative abundance (RA: %) of cyanobacteria species or above taxonomies was calculated as RA = 100 × (the number of cDNA sequences/the total cDNA sequences) for each library. For designing qPCR primers, the cDNA sequences associated with nitrogenase and phosphorus transport were retrieved. Fold changes were defined as (RA_date_ − RA_June3_)/RA_June3_, where RA_date_ indicates the RA at a specific during-bloom date and RA_June3_ indicates the RA at the pre-bloom date. Pearson correlations, multiple comparison tests, and linear analysis under PROC GLM were performed using SAS 9.4 (SAS Institute Inc., Cary, NC).

### qPCR and RT-qPCR

To validate the RNA-Seq data, real-time quantitative polymerase chain reaction (qPCR) assays targeting the main cyanobacteria involved in nitrogen and phosphorus metabolism were carried out using the sequences retrieved from RNA-Seq libraries. Transcripts targeting saxitoxin and MC genes were also amplified. The sequences and parameters of the assays are listed in Supplementary Table S1. For reverse transcription qPCR (RT-qPCR), total RNA was reverse transcribed to cDNA using the high-capacity cDNA reverse transcription kit (Life Technologies). The qPCR reaction mixtures (20 μL) contained 10 μL 2× qPCR SYBR Green Master Mix (Applied Biosystems, Foster City, CA), 0.2 μM primers (final concentration), and 2 μL of template cDNA. Initial cDNA treatment consisted of 50 °C for 2 min with uracil-N-glycosylase to prevent carryover contamination, then 95 °C for 10 min for cDNA denaturing. The following quantification cycling protocol was used with the QuantStudio 6 Flex system (Life Technologies): 40 cycles of 15 sec at 95 °C, 30 sec at 60 °C, and 30 sec at 72 °C. Following these cycles, a final hold at 72 °C was performed for 5 min. The cDNA copies for targeted genes were quantified relative to a standard curve for plasmids containing the target gene inserts as described previously^34^. Standards were made from serial dilutions of plasmids in nuclease-free water; 2 µL of each dilution was added to the 20-µL qPCR mixtures, providing a range of gene targets containing between 1 and 10^6^ gene copies µL^−1^. Each qPCR plate contained triplicate six-point standard curves with values ranging from 10^1^ to 10^9^ copies per reaction. Each cDNA sample was assayed for potential qPCR inhibitors with 10-fold dilution versus original cDNA. The least-square linear regressions of log_10_ target gene copies versus the cycle threshold were used to quantify the target gene copies in each sample. Copy numbers were also multiplied by a dilution factor and a water-filtering factor to present data as gene copies per liter of filtered lake water.

### Water parameters

Surface water temperatures were measured at sampling. Nutrients (nitrogen: total nitrogen [TN], total ammonium [TNH4], total nitrate [TNO3] and total nitrite [TNO2]; phosphorus: total phosphorus [TP] and total reactive phosphorus [TRP]) were measured using the Latchat Quickchem 8000, Flow Injection Analysis, Autoanalyzer (Hach Co., Loveland, CO) according to the manufacturer’s instruction.

## Supplementary information

supplemental document

## Data Availability

All data generated or analyzed during this study are included in this published article and its Supplementary Information files.
