# Supplementary material for: Nitrogen–phosphorus-associated metabolic activities during the development of a cyanobacterial bloom revealed by metatranscriptomics
Source: Sci Rep. 2019 Feb 21;9:2480. doi: 10.1038/s41598-019-38481-2 (PMC6385219; doi:10.1038/s41598-019-38481-2)
Supplement: Supplementary file 1 — supplemental document [file 41598_2019_38481_MOESM1_ESM.pdf]

Nitrogen–phosphorus-associated metabolic activities during the development of a cyanobacterial bloom revealed by metatranscriptomics

Jingrang Lu*[1], Bo Zhu[2], Ian Struewing[3], Ning Xu[2], & Shunshan Duan[2]

[1]U.S. Environmental Protection Agency Office of Research and Development, Cincinnati, OH, USA

[2]Institute of Hydrobiology, Jinan University, Guangzhou, Guangdong, China

[3]Pegasus Technical Services, Cincinnati, OH, USA

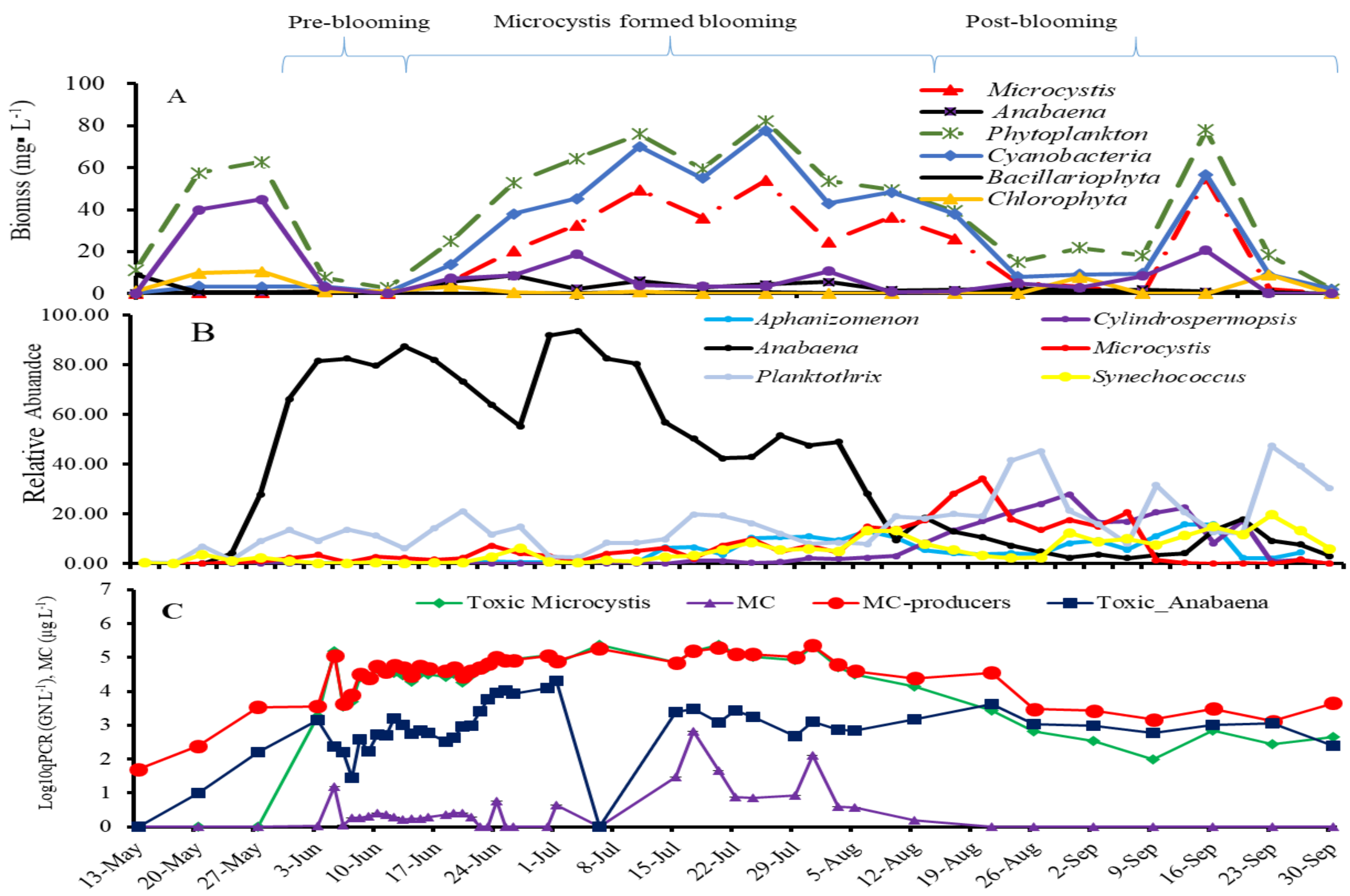

Fig. S1. Demonstration of temporal variations during the periods of pre-blooming, blooming and post-blooming according to (A) of the biomass of total phytoplankton, cyanobacteria and *Microcystis*, based on the data of algal counts using microscope (Chen et al. 2017), (B) main cyanobacterial genera and (C) total microcystin producers, toxic *Microcystis* and toxic *Anabaena* as measured by qPCR and total microcystin detected using LC-MS/MS for the same sampling site as in this study in Harsha Lake in 2015

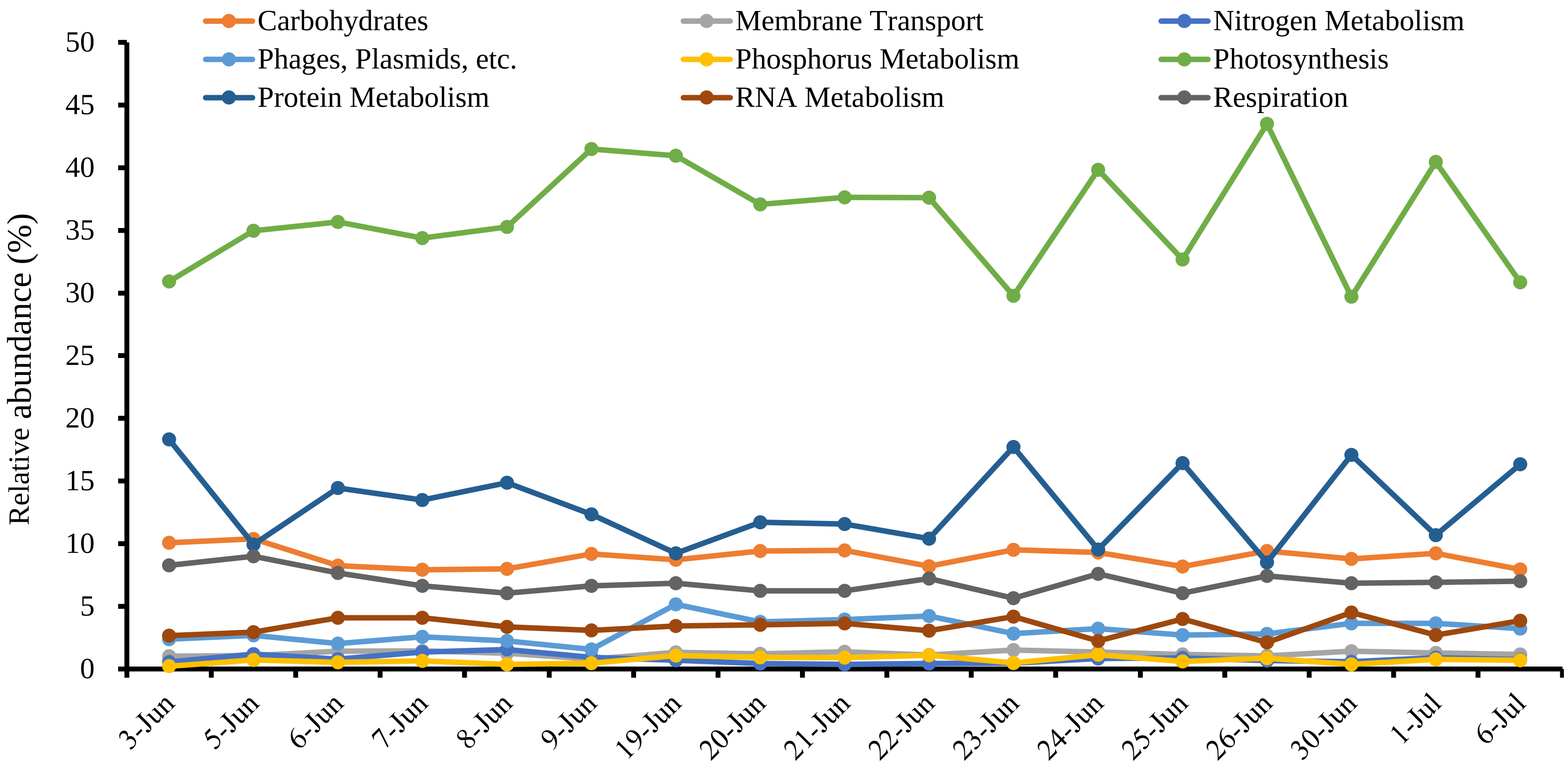

Fig. S2. Relative abundance of major functional transcriptome categories

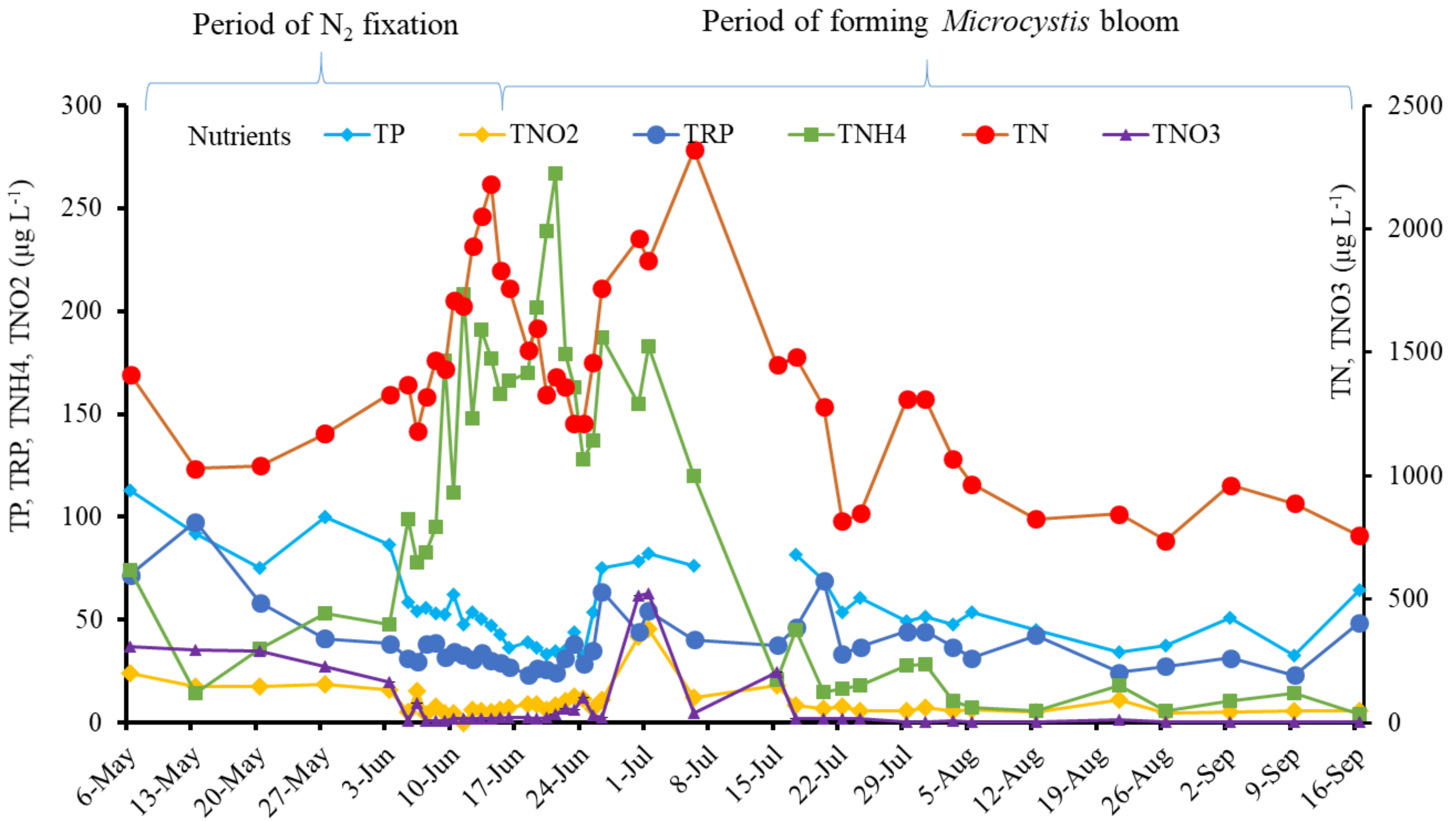

Fig. S3. Variations of nutrients measured in total (T) for nitrogen (TN), nitrate (TNO3), nitrite (TNO2), amonium (TNH4), phosphorus (TP) and reactive phosphorus (TRP) in Harsha Lake

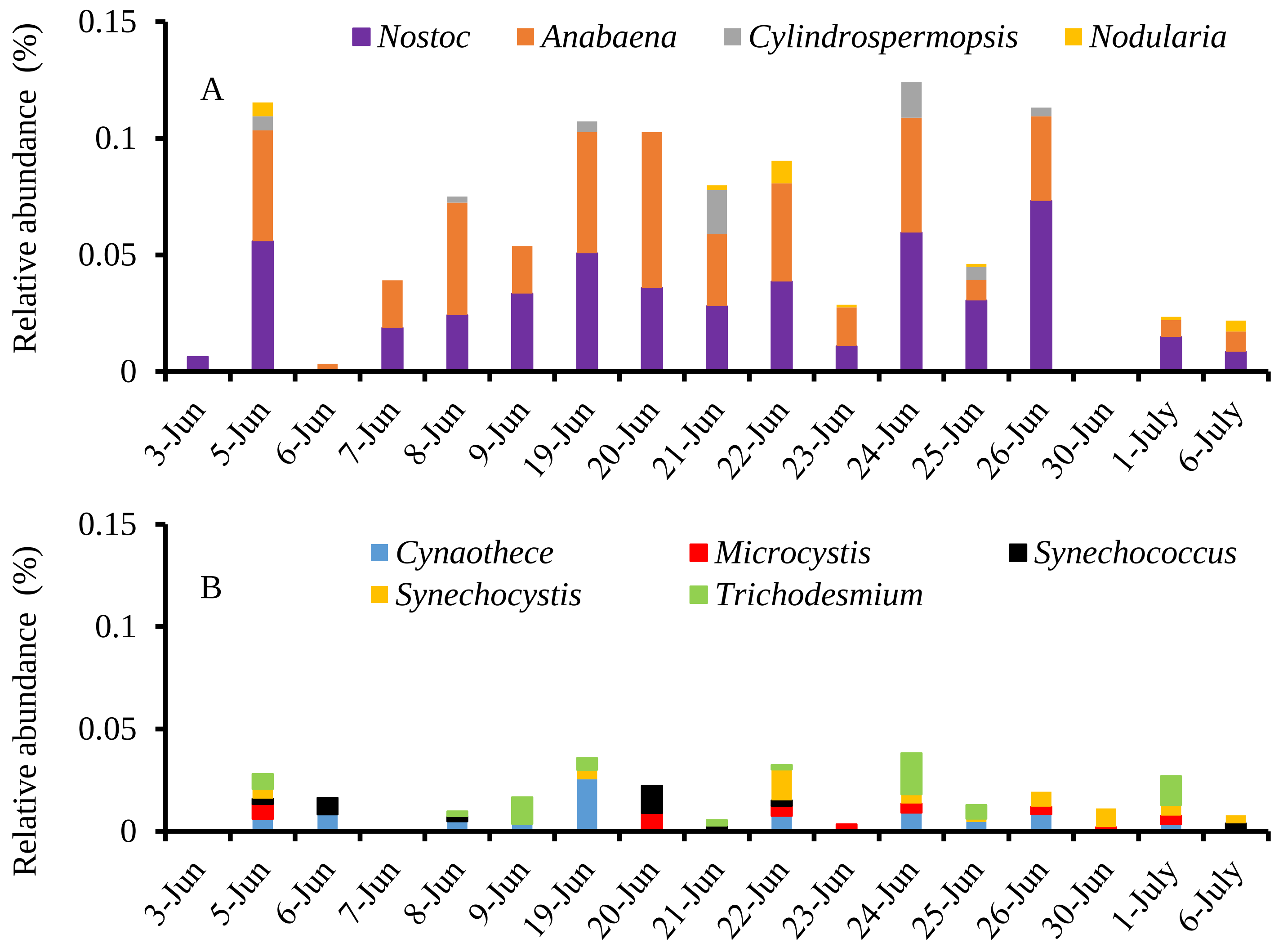

Fig. S4 Relative abundances of sequences similar to phosphorus metabolic pathway: (A) major genera within *Nostocales*, (B) major genera within Chroococcales and Oscillatoriales.

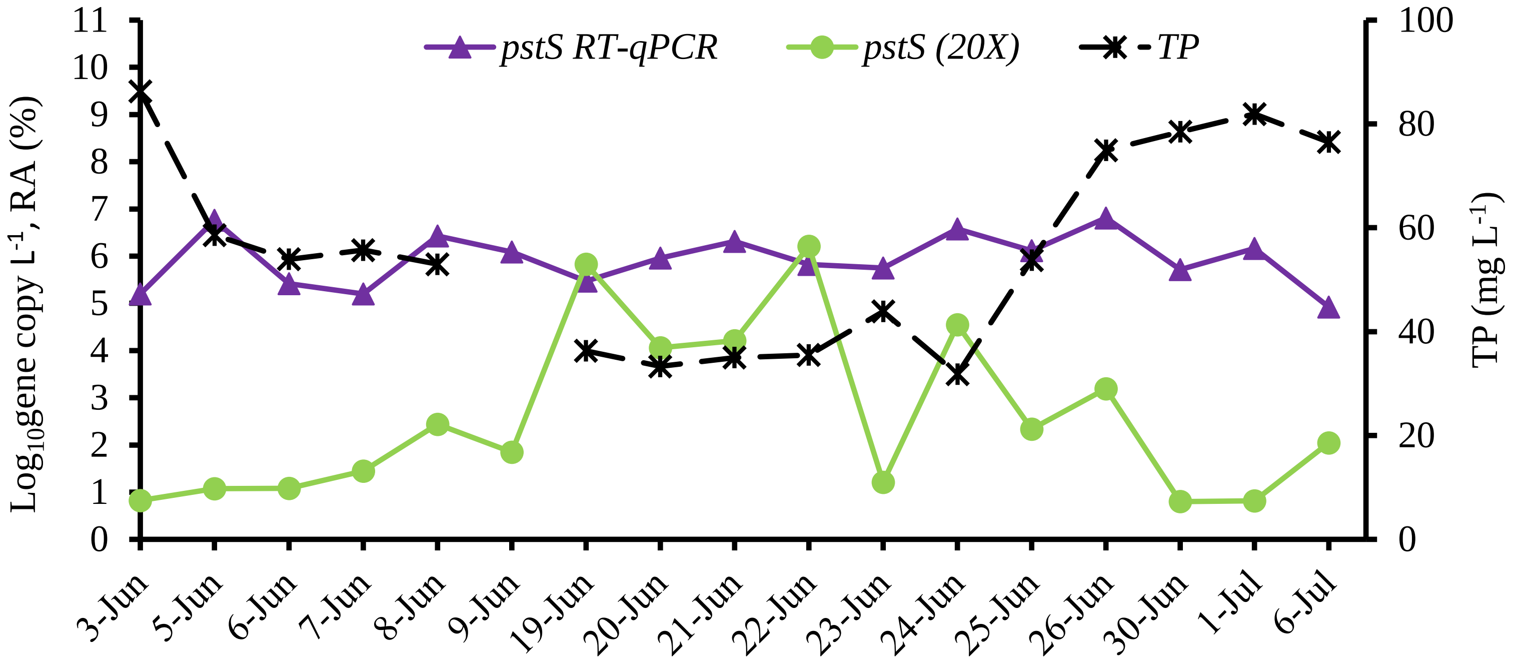

Fig. S5. -Fold expression profiles of genes associated with phosphorus metabolism as measured by qPCR data.

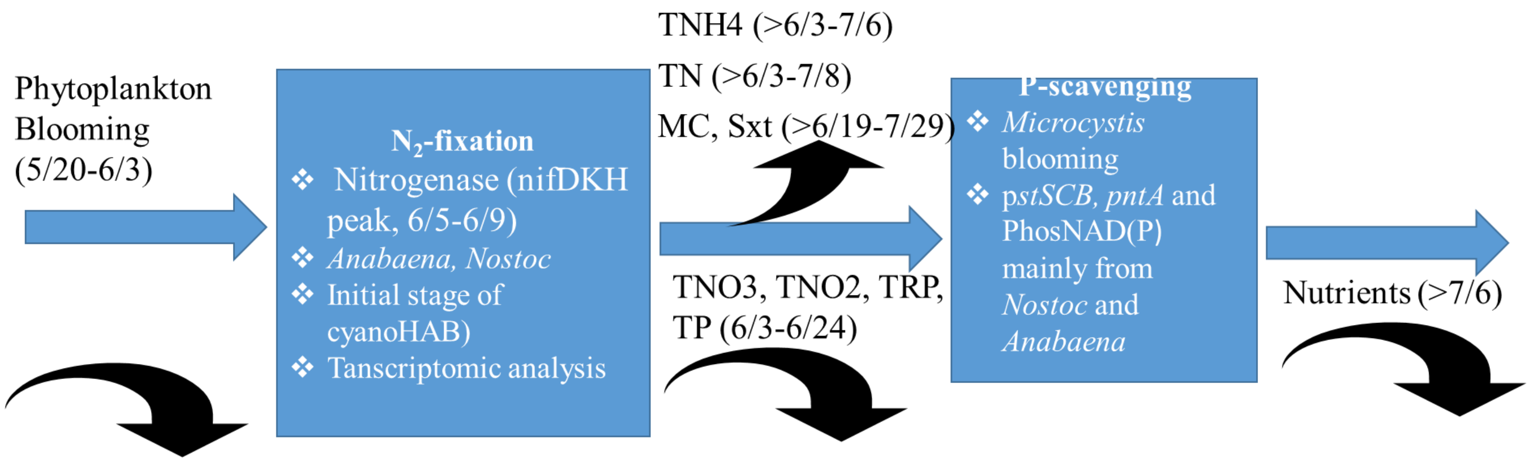

Fig. S6. Diagram of N_2-fixation and P-scavenge

Table S1. qPCR Primers used in this study

Primer	Sequence (5'→ 3')	Target	Product length	Tm (℃)	Limit of detection (copy number reaction^{-1})	Reference
mcyG-67F<br>mcyG-310R	CAA CCC AAC AGG TTC TTA AAG C<br>TGA GGC AAG GTT TCC TCT TG	*Microcystis* mcyG	244	60	10	Ngwa et al., 2012
stxA5f<br>stxArr	CGC TAT ACC CAC GGA TTT GTT<br>GGG ATC AGC AGT AGT CCA TCT A	Saxitoxin	120	60	10	This study
pstSf3<br>pstSr3	TGG AAT GTT ACC AGC AGG AAT AA<br>AGT GCT GCT TGA CGT AAA CT	A Flos Aq, AFA	110	60	10	This study
nif_anaF6<br>nif_anaR6	ATG CCT ATC CGT GAA GGT AAA G<br>CCA CCG GAG TGA GCA TAT TT	Ana, Nos, Cylin	86	60	10	This study
nif_nostF3<br>nif_nostR3	ATC GTT CAA CAC GCA GAA TTG<br>TCA TCC ATT TCG ATA GGT GTG G	Ana, Nos, Cyl	90	60	10	This study

Table S2, Summary of libarary sequences

Item	Quantity
Library number	17
Total (read)	15,899,228
Average leangth (bp)	199.02 ± 75.64
Average GC content (%)	47.47 ± 6.71
Range per library (read)	21,326 - 499,108
Average (read)	274,124
Total predicted protein features (read)	741,631
Annoted (read) and percentage (%)	277015, 37.35

Table S3. cDNA sequence hit[1] nitrogenase gene from the genome data of N2-fixation cyanobacterial isolates

Accession No	Gene	NCBI Organism	Order	Reference
YP_325033	twin-arginine translocation pathway signal protein	Anabaena variabilis ATCC 29413	Nostocales	Schrautemeier et al. 1995
YP_325034	nitrate transport permease	Anabaena variabilis ATCC 29413	heterocystous	
YP_320669	hypothetical protein	Anabaena variabilis ATCC 29413		
YP_323462	ammonium transporter	Anabaena variabilis ATCC 29413		
YP_324738	nitrogenase cofactor biosynthesis protein NifB	Anabaena variabilis ATCC 29413		
YP_325035	nitrate transport ATP-binding subunits C and D	Anabaena variabilis ATCC 29413		
YP_320658	nitrogen regulatory protein P-II	Anabaena variabilis ATCC 29413		
YP_324416	nitrogenase reductase	Anabaena variabilis ATCC 29413		
YP_321394	twin-arginine translocation pathway signal protein	Anabaena variabilis ATCC 29413		
YP_324417	nitrogenase molybdenum-iron protein subunit alpha	Anabaena variabilis ATCC 29413		
YP_323460	ammonium transporter	Anabaena variabilis ATCC 29413		
YP_001864115	nitrogenase molybdenum-iron protein beta chain NifK	Nostoc punctiforme PCC 73102		Summers et al. 1995
YP_001865159	nitrite transporter NrtP	Nostoc punctiforme PCC 73102		
YP_001865160	ferredoxin-nitrite reductase	Nostoc punctiforme PCC 73102		
YP_001864139	Fe-S cluster assembly protein NifU	Nostoc punctiforme PCC 73102		
NP_485035	ammonium transporter	Nostoc sp. PCC 7120		
NP_484652	nitrate transport nitrate-binding protein	Nostoc sp. PCC 7120		
NP_487398	3-hydroxyacid dehydrogenase	Nostoc sp. PCC 7120		
NP_484656	nitrate reductase	Nostoc sp. PCC 7120		
NP_485557	nitrogen fixation protein	Nostoc sp. PCC 7120		
NP_484654	nitrate transport ATP-binding protein	Nostoc sp. PCC 7120		

ZP_06306629	Glutamate-1-semialdehyde-	Cylindrospermopsis raciborskii CS-505]		Willis et al. 2016
ZP_06306743	Nitrite/sulfite reductas	Cylindrospermopsis raciborskii CS-505]		
ZP_06306629	Glutamate-1-semialdehyde-	Cylindrospermopsis raciborskii CS-505]		
ZP_06306741	Nitrate transport permease	Cylindrospermopsis raciborskii CS-505]		
ZP_06306839	Nitrate transport ATP-binding subunits C and D	Cylindrospermopsis raciborskii CS-505]		
ZP_01631054	UDP-N-acetylmuramoylalanyl-D-glutamate--	Nodularia spumigena CCY9414]		Vintila & El-Shehawy 2017
ZP_01630726	Nitrogen regulatory protein P-II (Gln	Nodularia spumigena CCY9414]		
ZP_01628441	nitrogen fixation protein	Nodularia spumigena CCY9414]		
ZP_01630828	glutamate--ammonia ligase	Nodularia spumigena CCY9414]		
ZP_01629158	folylpolyglutamate synthase	Nodularia spumigena CCY9414]		
ZP_03272006	nitrogen-fixing NifU domain protein	Arthrospira maxima CS-328	Oscillatoriales	Carrieri et al. 2011
ZP_03274031	Mo-dependent nitrogenase family protein	Arthrospira maxima CS-328	non-heterocystous	
ZP_06383243	nitrogen-fixing NifU domain protein	Arthrospira platensis str. Paraca	diazotroph	
ZP_06383913	Mo-dependent nitrogenase family protein	Arthrospira platensis str. Paraca		
ZP_06384291	nitrogen regulatory protein P-I: lnB	Arthrospira platensis str. Paraca; Arthrospira maxima CS-328		
YP_001801977	nitrogenase molybdenum-iron protein subunit alpha	Cyanothece sp. ATCC 51142		
YP_002380475	Mo-dependent nitrogenase family protein	Cyanothece sp. PCC 7424		Carrieri et al. 2011
YP_002483083	nitrogenase iron protein	Cyanothece sp. PCC 7425		
YP_002483442	nitrogen regulatory protein P-II	Cyanothece sp. PCC 7425		
YP_002483085	nitrogenase molybdenum-iron protein beta chain	Cyanothece sp. PCC 7425		
YP_003886770	Mo-dependent nitrogenase family protein	Cyanothece sp. PCC 7822		
YP_001801977	nitrogenase molybdenum-iron protein subunit alpha	Cyanothece sp. ATCC 51142		
YP_002380475	Mo-dependent nitrogenase family protein	Cyanothece sp. PCC 7424		
YP_002483083	nitrogenase iron protein	Cyanothece sp. PCC 7425		
YP_002483442	nitrogen regulatory protein P-II	Cyanothece sp. PCC 7425		
YP_002483085	nitrogenase molybdenum-iron protein beta chain	Cyanothece sp. PCC 7425		

YP_003886770	Mo-dependent nitrogenase family protein	Cyanothece sp. PCC 7822		
YP_002371988<br>YP_003137548	nitrogenase molybdenum-iron protein alpha chain;<br>nitrogenase molybdenum-iron protein alpha chain	Cyanothece sp. PCC 8801;<br>Cyanothece sp. PCC 8802		Carrieri et al.<br>2011
YP_003137727<br>YP_002372168	Mo-dependent nitrogenase family protein;<br>Mo-dependent nitrogenase family protein	Cyanothece sp. PCC 8802;<br>Cyanothece sp. PCC 8801		
YP_003137542<br>YP_002371982	nitrogenase cofactor biosynthesis protein NifB;<br>nitrogenase cofactor biosynthesis protein NifB	Cyanothece sp. PCC 8802 ;<br>Cyanothece sp. PCC 8801		
YP_003137549<br>YP_002371989	nitrogenase molybdenum-iron protein beta chain;<br>nitrogenase molybdenum-iron protein beta chain	Cyanothece sp. PCC 8802;<br>Cyanothece sp. PCC 8801		
YP_003139076<br>YP_002372847	protein of unknown function nitrogen fixation;<br>protein of unknown function nitrogen fixation	Cyanothece sp. PCC 8802;<br>Cyanothece sp. PCC 8801		
YP_723595	Mo-dependent nitrogenase-like	Trichodesmium erythraeum IMS101		Dyhrman et<br>al.<br>2006
YP_723937	ammonium transporter	Trichodesmium erythraeum IMS101		
YP_720396	glutamate synthase (ferredoxin)	Trichodesmium erythraeum IMS101		
YP_720911	nitrite transporter	Trichodesmium erythraeum IMS101		
NP_441331	global nitrogen regulator	Synechocystis sp. PCC 6803	Chroococcales	Berman-Frank<br>et al. 2003
ZP_00516387	Nitrogenase molybdenum-iron protein alpha chain:<br>Nitrogenase component	Crocosphaera watsonii WH 8501	non-<br>heterocystous	Dron et al.<br>2012
YP_001659015	ammonium/methylammonium permease	Microcystis aeruginosa NIES-843	diazotroph	
YP_001656830,<br>CP012375	Mo-dependent nitrogenase-like	Microcystis aeruginosa NIES-<br>2481;<br>NIES-843		
YP_001656830	Mo-dependent nitrogenase-like	Microcystis aeruginosa NIES-843		
YP_001656527	periplasmic beta-type carbonic anhydrase	Microcystis aeruginosa NIES-843		
YP_001660760	nitrogen regulatory protein P-II	Microcystis aeruginosa NIES-843		
YP_001656504	NADH-dependent glutamate synthase small subunit	Microcystis aeruginosa NIES-843		
YP_001656491	nitrate/nitrite transport system ATP-binding protein	Microcystis aeruginosa NIES-843		
YP_001656941	glutamate--ammonia ligase	Microcystis aeruginosa NIES-843		
YP_001660812	hydroxylamine reductase	Microcystis aeruginosa NIES-843		
YP_001657925	ferredoxin-dependent glutamate synthase	Microcystis aeruginosa NIES-843		
YP_001655197	global nitrogen regulator Ycf28	Microcystis aeruginosa NIES-843		

YP_399340	nitrogen regulatory protein P-II;	Synechococcus elongatus PCC 7942;		
YP_171902	nitrogen regulatory protein P-II	Synechococcus elongatus PCC 6301	Synechococcales	Fay 1992
YP_476682	nitrogenase molybdenum-iron protein beta chain	Synechococcus sp. JA-2-3B"a(2-13)	non-heterocystous	
YP_478892	nitrogen regulatory protein P-II;	Synechococcus sp. JA-2-3B"a(2-13);		
YP_474937	nitrogen regulatory protein P-II	Synechococcus sp. JA-3-3Ab	diazotroph	

[1]cut-off values of a maximum e-value of 1e-5, a minimum identity of 60% and a minimum alignment length of 15 measured in amino acid for protein and base pair for RNA databases

References

Schrautemeier, B., Neveling, U., & Schmitz, S. (1995). Distinct and differently regulated Mo-dependent nitrogen-fixing systems evolved for heterocysts and vegetative cells of Anabaena variabilis ATCC 29413: characterization of the fdxH1/2 gene regions as part of the nif1/2 gene clusters. *Molecular microbiology*, *18*(2), 357-369.

Summers, M. L., Wallis, J. G., Campbell, E. L., & Meeks, J. C. (1995). Genetic evidence of a major role for glucose-6-phosphate dehydrogenase in nitrogen fixation and dark growth of the cyanobacterium Nostoc sp. strain ATCC 29133. *Journal of bacteriology*, *177*(21), 6184-6194.

Willis, A., Chuang, A. W., & Burford, M. A. (2016). Nitrogen fixation by the diazotroph Cylindrospermopsis raciborskii (Cyanophyceae). *Journal of phycology*, *52*(5), 854-862.

Vintila, S., & El-Shehawy, R. (2007). Ammonium ions inhibit nitrogen fixation but do not affect heterocyst frequency in the bloom-forming cyanobacterium Nodularia spumigena strain AV1. *Microbiology*, *153*(11), 3704-3712.

Carrieri, D., Wawrousek, K., Eckert, C., Yu, J., & Maness, P. C. (2011). The role of the bidirectional hydrogenase in cyanobacteria. *Bioresource technology*, *102*(18), 8368-8377.

Dyhrman, S. T., Chappell, P. D., Haley, S. T., Moffett, J. W., Orchard, E. D., Waterbury, J. B., & Webb, E. A. (2006). Phosphonate utilization by the globally important marine diazotroph Trichodesmium. *Nature*, *439*(7072), 68.

Berman-Frank, I., Lundgren, P., & Falkowski, P. (2003). Nitrogen fixation and photosynthetic oxygen evolution in cyanobacteria. *Research in microbiology*, *154*(3), 157-164.

Dron, A., Rabouille, S., Claquin, P., Le Roy, B., Talec, A., & Sciandra, A. (2012). Light–dark (12: 12) cycle of carbon and nitrogen metabolism in Crocosphaera watsonii WH8501: relation to the cell cycle. *Environmental microbiology*, *14*(4), 967-981.

Fay, P. (1992). Oxygen relations of nitrogen fixation in cyanobacteria. *Microbiological reviews*, *56*(2), 340-373.

Table S4 Correlations between the abundances of genera associated with nitrogenase and phosphorus transportation (P<0.10 with blue highlights), and among those associated with nitrogenase or phosphorus transportation, respectively (P<0.10 with yellow highlights)

	***Cynaothece*, P**	***Cynaothece*, N**	***Microcystis*, P**	***Microcystis*, N**	***Synechococcus*, P**	***Synechococcus*, N**	***Synechocystis*, P**	***Synechocystis*, N**	***Trichodesmium*, P**	***Trichodesmium*, N**	***Nostoc*, P**	***Nostoc*, N**	***Anabaena*, P**	***Anabaena*, N.**	***Cylindrospermopsis*, P**	***Cylindrospermopsis* N**	***Nodularia*, P**	***Nodularia*, N**
Cynaothece, P	1.000	0.001	-0.048	-0.091	-0.163	0.627 (P=0.007)	0.270	0.176	0.277	-0.199	0.523 (P=0.031)	0.142	0.409	0.222	0.153	-0.086	-0.026	-0.049
Cynaothece, N	0.001	1.000	-0.006	0.425 (P=0.089)	-0.083	0.183	-0.182	-0.149	0.098	-0.088	0.169	0.906 (P=0.000)	0.275	0.838 (P=0.000)	-0.065	0.686 (P=0.002)	0.136	0.781 (P=0.000)
Microcystis, P	-0.048	-0.006	1.000	0.452 (P=0.068)	0.432	-0.300	0.357	0.201	0.165	-0.198	0.453 (P=0.068)	-0.040	0.570 (P=0.017)	0.024	0.002	-0.073	0.327	-0.097
Microcystis, N	-0.091	0.425 (P=0.089)	0.452 (P=0.000)	1.000	-0.092	-0.223	0.200	-0.114	0.098	-0.114	0.063	0.282	0.005	0.363	-0.021	0.169	0.310	0.201
Synechococcus, P	-0.163	-0.083	0.432	-0.092	1.000	-0.079	-0.209	-0.151	-0.332	0.125	-0.105	0.034	0.347	0.134	-0.134	0.319	0.063	0.025
Synechococcus, N	0.627 (P=0.007)	0.183	-0.300	-0.223	-0.079	1.000	0.019	-0.123	-0.047	-0.123	0.199	0.324	0.450 (P=0.070)	0.304	0.266	0.197	-0.026	0.133
Synechocystis, P	0.270	-0.182	0.357	0.200	-0.209	0.019	1.000	0.073	0.059	0.038	0.287	-0.227	0.108	-0.224	-0.087	-0.387	0.616	-0.301
Synechocystis, N	0.176	-0.149	0.201	-0.114	-0.151	-0.123	0.073	1.000	0.663 (P=0.004)	-0.063	0.367	-0.165	0.270	-0.122	0.547 (P=0.023)	-0.194	-0.145	0.017
Trichodesmium, P	0.277	0.098	0.165	0.098	-0.332	-0.047	0.059	0.663 (P=0.004)	1.000	-0.185	0.395	0.095	0.171	0.170	0.411	0.051	-0.053	-0.071
Trichodesmium, N	-0.199	-0.088	-0.198	-0.114	0.125	-0.123	0.038	-0.063	-0.185	1.000	-0.240	-0.171	-0.224	-0.187	-0.152	-0.099	0.298	-0.152
Nostoc, P	0.523 (P=0.031)	0.169	0.453	0.063	-0.105	0.199	0.287	0.367	0.395	-0.240	1.000	0.204	0.741 (P=0.000)	0.194	0.450	0.018	0.128	-0.013
Nostoc, N	0.142	0.906 (P=0.000)	-0.040	0.282	0.034	0.324	-0.227	-0.165	0.095	-0.171	0.204	1.000	0.328	0.964 (P=0.000)	-0.051	0.835 (P=0.000)	-0.023	0.649 (P=0.005)
Anabaena, P	0.409	0.275	0.570 (P=0.017)	0.005	0.347	0.450 (P=0.070)	0.108	0.270	0.171	-0.224	0.741 (P=0.000)	0.328	1.000	0.318	0.335	0.207	0.138	0.184
Anabaena, N	0.222	0.838 (P=0.000)	0.024	0.363	0.134	0.304	-0.224	-0.122	0.170	-0.187	0.194	0.964 (P=0.000)	0.318	1.000	0.008	0.866 (P=0.000)	0.009	0.582 (P=0.014)
Cylindrospermopsis, P	0.153	-0.065	0.002	-0.021	-0.134	0.266	-0.087	0.547 (P=0.023)	0.411	-0.152	0.450 (P=0.070)	-0.051	0.335	0.008	1.000	0.120	-0.018	-0.021
Cylindrospermopsis, N	-0.086	0.686 (P=0.002)	-0.073	0.169	0.319	0.197	-0.387	-0.194	0.051	-0.099	0.018	0.835 (P=0.000)	0.207	0.866 (P=0.000)	0.120	1.000	-0.042	0.425 (P=0.089)
Nodularia, P	-0.026	0.136	0.327	0.310	0.063	-0.026	0.616 (P=0.008)	-0.145	-0.053	0.298	0.128	-0.023	0.138	0.009	-0.018	-0.042	1.000	-0.070
Nodularia, N	-0.049	0.781 (P=0.000)	-0.097	0.201	0.025	0.133	-0.301	0.017	-0.071	-0.152	-0.013	0.649 (P=0.005)	0.184	0.582 (P=0.014)	-0.021	0.425 (P=0.089)	-0.070	1.000
